# High-resolution diffusion kurtosis imaging at 3T enabled by advanced post-processing

**DOI:** 10.3389/fnins.2014.00427

**Published:** 2015-01-07

**Authors:** Siawoosh Mohammadi, Karsten Tabelow, Lars Ruthotto, Thorsten Feiweier, Jörg Polzehl, Nikolaus Weiskopf

**Affiliations:** ^1^Wellcome Trust Centre for Neuroimaging, UCL Institute of Neurology, University College LondonLondon, UK; ^2^Department of Systems Neuroscience, University Medical Center Hamburg-EppendorfHamburg, Germany; ^3^Stochastic Algorithms and Nonparametric Statistics, Weierstrass Institute for Applied Analysis and StochasticsBerlin, Germany; ^4^Department of Earth, Ocean and Atmospheric Sciences, The University of British ColumbiaVancouver, BC, Canada; ^5^Healthcare Sector, Siemens AGErlangen, Germany

**Keywords:** DTI, DKI, diffusion kurtosis, gray matter, high-resolution, multi-shell dMRI, eddy current and motion artifacts, adaptive smoothing

## Abstract

Diffusion Kurtosis Imaging (DKI) is more sensitive to microstructural differences and can be related to more specific micro-scale metrics (e.g., intra-axonal volume fraction) than diffusion tensor imaging (DTI), offering exceptional potential for clinical diagnosis and research into the white and gray matter. Currently DKI is acquired only at low spatial resolution (2–3 mm isotropic), because of the lower signal-to-noise ratio (SNR) and higher artifact level associated with the technically more demanding DKI. Higher spatial resolution of about 1 mm is required for the characterization of fine white matter pathways or cortical microstructure. We used restricted-field-of-view (rFoV) imaging in combination with advanced post-processing methods to enable unprecedented high-quality, high-resolution DKI (1.2 mm isotropic) on a clinical 3T scanner. Post-processing was advanced by developing a novel method for Retrospective Eddy current and Motion ArtifacT Correction in High-resolution, multi-shell diffusion data (REMATCH). Furthermore, we applied a powerful edge preserving denoising method, denoted as multi-shell orientation-position-adaptive smoothing (msPOAS). We demonstrated the feasibility of high-quality, high-resolution DKI and its potential for delineating highly myelinated fiber pathways in the motor cortex. REMATCH performs robustly even at the low SNR level of high-resolution DKI, where standard EC and motion correction failed (i.e., produced incorrectly aligned images) and thus biased the diffusion model fit. We showed that the combination of REMATCH and msPOAS increased the contrast between gray and white matter in mean kurtosis (MK) maps by about 35% and at the same time preserves the original distribution of MK values, whereas standard Gaussian smoothing strongly biases the distribution.

## Introduction

Conventional diffusion tensor imaging (DTI) has become an important tool in clinical research and neuroscience (e.g., Zatorre et al., 2012; Filippi and Rocca, 2013). The interest in DTI is mainly elicited by two applications: DTI-based indices, such as the fractional anisotropy (FA) or the mean diffusivity (MD), are sensitive to functional differences in healthy subjects (e.g., due to handedness, Büchel et al., 2004; Mohammadi et al., 2012a) and to disease-related brain-tissue alterations (e.g., Kovac et al., 2009; Meinzer et al., 2010; Warnecke et al., 2010; Duning et al., 2011; Freund et al., 2013a). Moreover, the linear DTI model is easy to implement and can be estimated robustly even in the presence of outliers (e.g., Mohammadi et al., 2013a,b).

However, DTI measures are difficult to relate to the cerebral gray and white matter (GM and WM) microstructure. In WM, for example, very different configurations of axon density, size and myelination may result in the same FA and MD (e.g., Jones et al., 2013). In GM, the interpretation of DTI is even more difficult, because diffusion anisotropy is generated by the distribution of neurites in general rather than solely by axonal properties (Jespersen et al., 2010).

To disentangle restricted diffusion in axons from other anisotropic diffusion mechanisms in dMRI (e.g., crossing fibers or other tissue compartments), beyond-tensor models have been introduced (e.g., see Assemlal et al., 2011 for a summary). Beyond Gaussian-diffusion-tensor models include for example: multi-compartment models such as the combined hindered and restricted model of diffusion (CHARMED, Assaf and Basser, 2005), neurite orientation dispersion and density imaging (NODDI, Zhang et al., 2012), the 4th-order tensor model for the double-wave-vector measurement (e.g., Lawrenz and Finsterbusch, 2013), multi-tensor models (e.g., Behrens et al., 2003, p. 203; Tabelow et al., 2012), or other beyond Gaussian-diffusion models (e.g., diffusion kurtosis imaging, DKI, Özarslan and Mareci, 2003; Jensen et al., 2005).

DKI is the most intuitive extension of the DTI model, because it quantifies the deviation from Gaussian diffusion, upon which DTI is based (Jensen and Helpern, 2010). It has methodological similarities to DTI and thus is straightforward to implement (e.g., Tabesh et al., 2011) i.e., can be approximated by a linear model (Jensen and Helpern, 2010). Furthermore, mean-kurtosis-like measures can be acquired in about 1 min scan time (Hansen et al., 2013).

Moreover, the kurtosis tensor metrics can be related to the intra-axonal volume fraction (see, e.g., Fieremans et al., 2010, 2011; De Santis et al., 2012), making DKI more specific to the cerebral microstructure than DTI. Following pathological alterations, significant differences in the kurtosis values were observed, which were not detected with DTI [e.g., grading gliomas (Van Cauter et al., 2012) or assessing stroke-related changes (Hui et al., 2012)]. In GM, high-resolution DKI has proved to be more specific to the extracellular volume than DTI (Jespersen et al., 2010). These new findings support the importance of DKI for characterizing (more specific) properties of WM and GM microstructure. GM-DKI might be particularly of interest for neuroscience and clinical research. For example, amyloid plaques in the cortex as found in Alzheimer's disease (see e.g., Meadowcroft et al., 2009) could lead to non-Gaussian diffusion and thus be particularly efficiently detected by DKI.

The main reason why DTI but not DKI is still regularly used in neuroscience and clinical research is that DKI is poorly conditioned and thus requires expert-knowledge to be robustly estimated (e.g., Tabesh et al., 2011; Veraart et al., 2011, 2013a,b; Tax et al., 2014). These issues also apply to all other beyond-tensor models (e.g., Assaf and Basser, 2005; Zhang et al., 2012) and reduce their practical usage in daily research. To robustly solve this poorly conditioned problem, data with different and higher diffusion-weightings (i.e., *b*-value, Basser et al., 1994) are acquired in addition to multiple diffusion directions (see e.g., Poot et al., 2010). The higher diffusion weighting results in a lower signal-to-noise ratio (SNR) in the DKI measurement. Since higher gradient amplitudes are required, motion and physiological noise artifacts (e.g., Kristoffersen, 2011; Mohammadi et al., 2013a,b), instrumental artifacts such as eddy currents (EC) (e.g., Andersson and Skare, 2002; Mohammadi et al., 2010), gradient inhomogeneities (e.g., Mohammadi et al., 2012c), or vibration artifacts (e.g., Hiltunen et al., 2006; Mohammadi et al., 2012b) are exacerbated compared to standard DTI. To compensate for these penalties and improve the reliability of DKI, usually multi-shell data with low spatial resolution are acquired (e.g., Wang et al., 2011; Hui et al., 2012; Van Cauter et al., 2012), although it has been shown that higher spatial resolution improves specificity to WM and GM microstructure (e.g., Jespersen et al., 2010; Heidemann et al., 2012; Mohammadi et al., 2013a). High-resolution DKI in GM of the human brain has not been reported at 3T, probably because a voxel size of about 1 mm isotropic resolution is imperative for dMRI to display GM properties (see e.g., McNab et al., 2013). One way to achieve a resolution of about 1 mm isotropic voxel size in a clinical acceptable time is to make use of restricted-field-of-view (rFoV) dMRI (e.g., Heidemann et al., 2010), which has not been used for the acquisition of multi-shell dMRI data yet.

Often no or only little post-processing is applied to improve results in DKI (e.g., Wang et al., 2011). If post-processing is applied, it relies on standard methods such as denoising based on Gaussian smoothing (e.g., Tabesh et al., 2011) or eddy current and motion artifact correction (e.g., Van Cauter et al., 2012), which are based on a single target (hereafter denoted as single-target registration method), although it is known that these methods might be insufficient for high-*b*-value dMRI data (see e.g., Nam and Park, 2011; Ben-Amitay et al., 2012).

We introduce three advanced post-processing and imaging methods to enable high-resolution DKI at 3T. First, we make use of rFoV imaging to acquire DKI at higher spatial resolution. Second, we introduce a novel eddy current and motion correction method specifically developed for low-SNR, high-resolution, multi-shell dMRI data. Third, we use our recently developed edge-preserving denoising method, hence denoted as multi-shell position-orientation-adaptive smoothing (msPOAS, Becker et al., 2012, 2014; Tabelow et al., 2014). msPOAS not only uses spatial and orientation information but also combines the information from different diffusion shells to increase the smoothing power (Becker et al., 2014; Tabelow et al., 2014). We demonstrate the importance of all three post-processing steps to enable high-fidelity high-resolution DKI at 1.2 mm in a group of five volunteers.

## Methods

### Subjects

Five healthy adult volunteers (1 female, 4 male, age: 32 ± 12) participated in the study approved by the local ethics committee after giving written informed consent.

### Data acquisition

Experiments were performed on a MAGNETOM Trio, a Tim system 3T scanner (Siemens AG, Healthcare Sector, Erlangen, Germany) operated with a radio-frequency (RF) body transmit coil and a 32-channel receive-only RF head coil.

#### dMRI

dMRI data were acquired with a prototype monopolar diffusion sequence (Morelli et al., 2010) using two different protocols: The first protocol (protocol 1), was acquired with very high spatial resolution and low SNR, using the following parameters: 1.2 mm slice thickness, with 10% inter-slice gap, 34 slices, phase oversampling 50%, 132 × 48 matrix, 156 × 58 mm FoV, 1.2 × 1.2 mm in-plane resolution, echo time of TE = 97 ms, volume repetition time of TR = 6100 ms, and two diffusion shells. In the first diffusion shell 100 diffusion weighted (DW) images with low diffusion weighting (*b* = 800 s/mm^2^) and isotropic distribution of diffusion directions as well as 10 evenly distributed *b* = 0 images were acquired. In the second diffusion shell 100 DW images were acquired with the same diffusion directions and high diffusion weighting (*b* = 2000 s/mm^2^) as well as 10 evenly distributed *b* = 0 images. To reduce dead time during scanning and use the same TE for both diffusion shells, the first shell was acquired with 7/8 Partial-Fourier (PF) imaging and the second shell with 5/8 PF imaging in the phase-encoding direction (anterior-posterior). The second protocol (protocol 2) was acquired at lower spatial resolution and higher SNR, using the parameters: 1.4 mm slice thickness, with 10% inter-slice gap, 34 slices, phase oversampling 50%, 110 × 42 matrix, 156 × 60 mm FoV, 1.4 × 1.4 mm in-plane resolution, echo time of TE = 107.6 ms, volume repetition time of TR = 6200 ms, 6/8 PF imaging in the phase-encoding direction (anterior-posterior), and three diffusion shells (*b* = 800, 2000, and 3000 s/mm^2^). For each shell, 70 DW images with isotropic distribution of diffusion directions were acquired, as well as seven evenly distributed *b* = 0 images. All diffusion directions were acquired according to Caruyer et al. (2013). The reduced FoV was enabled by using two RF saturation pulses (Heidemann et al., 2009), suppressing signal from tissue outside the FoV. The total acquisition time was 22.34 min and 24.12 min for protocol 1 and 2, respectively. dMRI with protocol 1 was aquired for all five subjects; for two subjects the second shell (i.e., the *b* = 800 s/mm^2^) was acquired in a different session (subject 2 and 3). These subjects were denoted as two-session subjects. Protocol 2 was acquired for subject 3 only. Note that no parallel imaging was used for protocol 1 and 2.

#### Magnetization transfer imaging

For each subject a whole-brain quantitative multi-parameter mapping (MPM) protocol (Dick et al., 2012; Weiskopf et al., 2013) was used to acquire high-definition 0.8 mm isotropic magnetization transfer saturation (MT) and longitudinal relaxation (R1) maps, which show an improved micro-structural definition of the cortex over standard T1-weighted anatomical scans, since they are quantitative and more specific (Dick et al., 2012; Weiskopf et al., 2013; Lutti et al., 2014). The protocol consisted of proton-density- (PD), longitudinal-relaxation-rate- (R1), and MT-weighted fast-low-angle-single-shot (FLASH) acquisitions as described in Weiskopf et al. (2013) using the following parameters: voxel size: 0.8 × 0.8 × 0.8 mm^3^, FoV 256 × 216 × 194 mm^3^, matrix 320 × 270 × 240, TR 23.7 ms, excitation flip angle: 6° (PDw) or 28° (T1w). Acquisition was accelerated by GRAPPA (with a parallel imaging factor of 2) in the phase encoding as well as by PF in the partition direction (with factor 6/8). To improve image quality (maximize SNR and minimize geometric distortion at the same time), eight gradient echoes were acquired with high readout bandwidth after each excitation pulse. The total scanning time of the MPM protocol was approximately 35 min. Quantitative maps were derived from the MPM protocol using MATLAB tools (The Mathworks Inc., Natick, MA, USA) implemented in a toolbox for voxel-based quantification (VBQ; Draganski et al., 2011; Weiskopf et al., 2013). The set of echoes for each of the three acquired weightings were then averaged to increase the SNR (11). The resulting PDw, T1w, and MTw volumes were used to calculate maps of MT and R1 as described previously (Weiskopf et al., 2013). The MT map is a semi-quantitative measure of the percentage loss of magnetization caused by a Gaussian RF pulse (4 ms duration, 220° nominal flip angle) applied 2 kHz off-resonance prior to non-selective excitation. This differs from the commonly used MT ratio (MTR; percentage reduction in steady state signal) by explicitly accounting for spatially varying T1 relaxation times and flip angles (Helms et al., 2008) and results in higher contrast in the brain than MTR (Helms et al., 2010). Additional minor corrections for flip angle inhomogeneity in the MT maps were applied as described in Weiskopf et al. (2013).

### Post-processing and tensor estimation pipelines

All proposed post-processing methods for high-resolution DKI were implemented in and performed with the Artifact Correction In Diffusion MRI (or ACID, www.diffusiontools.com) toolbox, which is an open-source add-on to SPM (Friston et al., 2006). All analysis steps were performed using SPM8 and SPM12 (http://www.fil.ion.ucl.ac.uk/spm, Friston et al., 2006), the ACID toolbox (www.diffusiontools.com), and in-house software written in MATLAB (version 7.11.0; Mathworks, Natick, MA, USA).

#### Eddy current and motion correction

The data were corrected for motion and eddy current artifacts using three different registration methods: (a) none, (b) an Eddy current and motion correction method for standard high-SNR DTI data (ECMOCO, for details see Mohammadi et al., 2010 and www.diffusiontools.com), and (c) a novel Retrospective Eddy current and Motion ArtifacT Correction method for High-resolution dMRI data (REMATCH).

#### REMATCH

**Figure 1:**
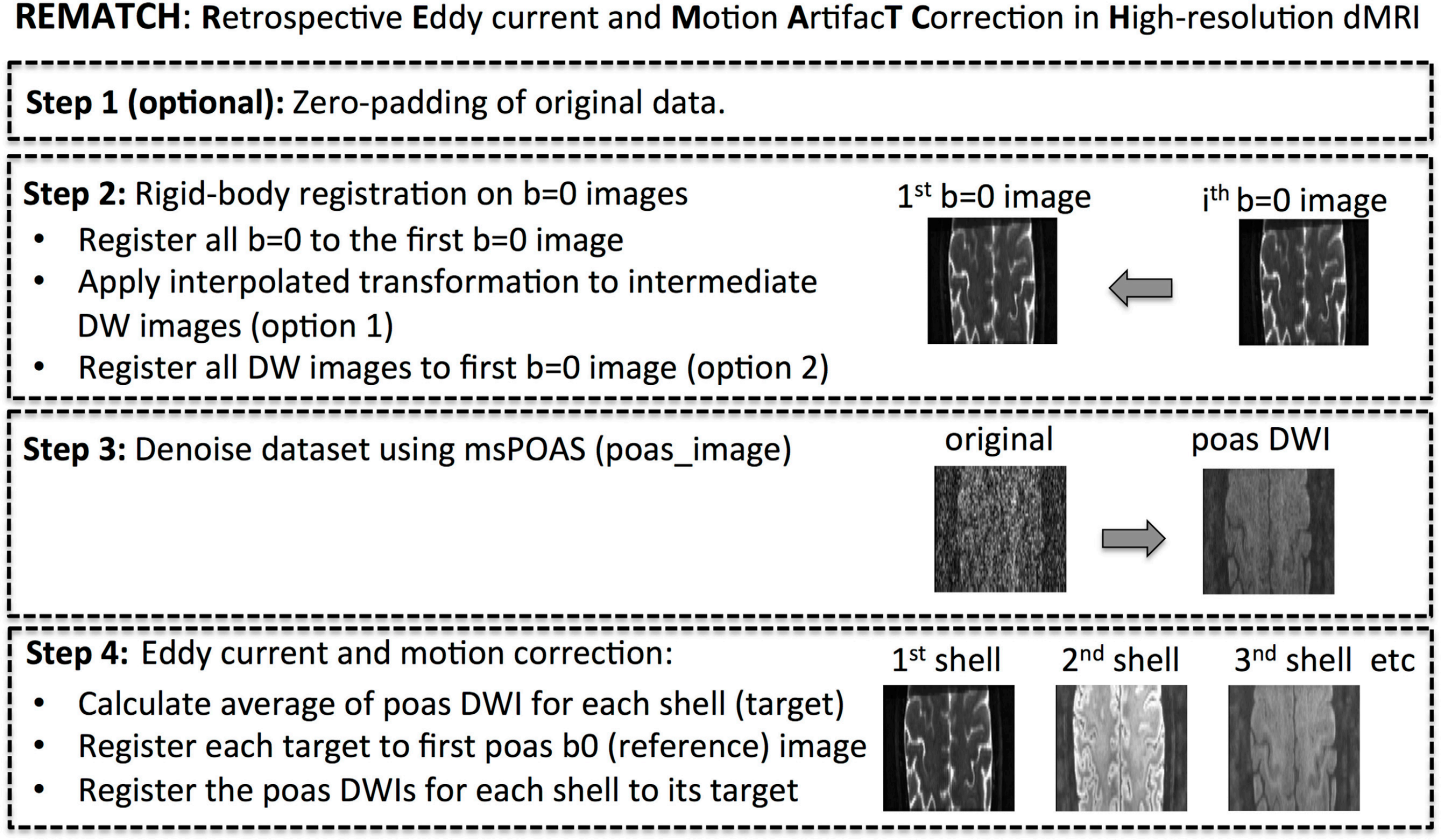
**Flowchart of proposed eddy current and motion correction method for high-resolution diffusion MRI data**.

Here, we introduce an eddy current and motion correction method that also works robustly for low-SNR, high-resolution data (see flowchart in Figure 1). To this end, REMATCH makes use of adaptive smoothing of the source and simulation of the target images to denoise the input data and increase robustness of the registration. In the first step, the original rFOV modulus image data were zero-padded by 20% to avoid reslicing artifacts (e.g., translation that moves part of the image outside the rFOV). In the second step, the *b* = 0 images were registered to the first *b* = 0 image using a rigid-body registration. Then, motion between *b* = 0 images was approximated linearly, i.e., each transformation parameter between adjacent *b* = 0 images was modeled by a linear function and the linear fit was applied to the intermediate DW images (option 1). Alternatively, e.g., if *b* = 0 images were not acquired throughout the DWI acquisition but at the beginning, the DWIs were registered to the *b* = 0 directly using a rigid-body registration (option 2). This step corrected for slow smooth movement. In the third step, each DW image was denoised using msPOAS. In the fourth and last step, the msPOAS-denoised DW images were registered to the corresponding target image, which was different for each shell. For this step, a nine-parameter affine transformation that corrects simultaneously for rigid-body motion and linear EC was used. The linear distortions due to EC were modeled as four affine distortions [(i) translation along the phase-encoding direction, (ii) scaling along the phase-encoding direction, (iii) in-plane shearing, and (iv) through-plane shearing], which have been derived elsewhere (Mohammadi et al., 2010). The target images were constructed by taking the median of the DW images within each shell. Finally, the transformation parameters from the REMATCH step 2 and 4 were applied to the original data. This method is implemented in the ACID toolbox.

#### Denoising

This step addressed the higher noise level in the data. Three different denoising methods were used: (a) none, (b) Gaussian smoothing as reported by Tabesh et al. (2011) (smoothing kernel: 3.375 × 3.375 × 3.375 mm^3^), and (c) multi-shell position-orientation-adaptive smoothing (msPOAS, Becker et al., 2012, 2014) with the settings recommended in Tabelow et al. (2014), i.e., kappa = 0.8, lamda = 10, and ncoils = 1.

#### Tensor estimation

This step estimated the diffusion and kurtosis tensor and their associated indices [i.e., FA, mean kurtosis (MK), and the root-mean-square of the model-fit error, ε], using the constrained least squares formulation as suggested in Tabesh et al. (2011). The quadratic program was solved individually for each voxel yielding many small-dimensional sub-problems that can be solved explicitly and in parallel. Each sub-problem was solved using a standard active-set method with a null-space method to improve robustness of the algorithm as described in Nocedal and Wright (2006).

### Analysis I: performance of REMATCH and comparison with ECMOCO

Two datasets with different SNR levels were used as pseudo ground truth to assess the performance of REMATCH: (a) the high-resolution diffusion dataset from subject 5 (acquired with protocol 1) with an average SNR level of about 16 and (b) simulated DW images calculated from the diffusion-tensor fit of (a), which can be treated as denoised data. The average SNR in the original dataset was estimated for the *b* = 0 s/mm2 images using the approach in Hutton et al. (2011). For the high-SNR dataset the difference between REMATCH and ECMOCO was expected to be small, whereas REMATCH was expected to outperform ECMOCO for lower SNR levels. Before perturbing the data, the original diffusion dataset showed relatively small eddy current and motion artifacts (assessed by visual inspection).

**Figure 2:**
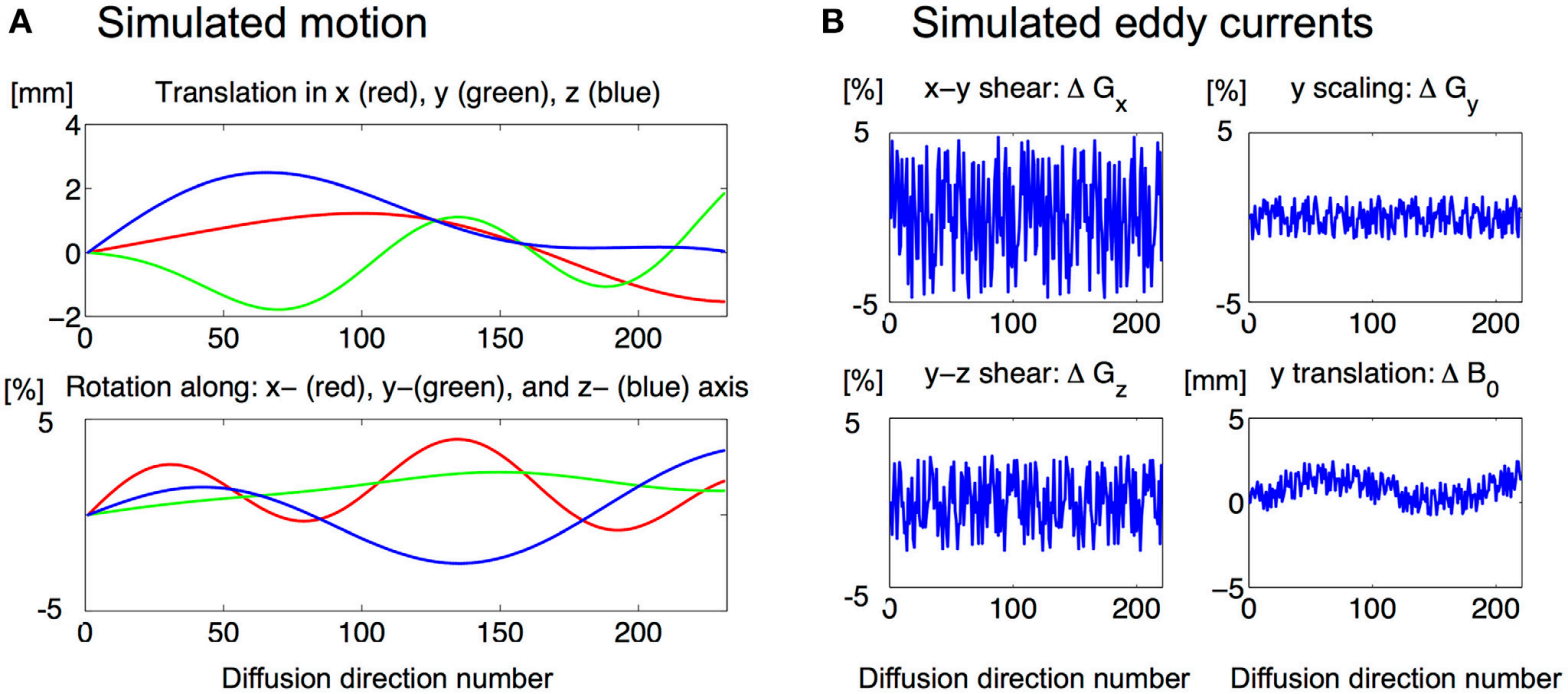
**One example of one set of (A) motion and (B) eddy current distortion parameters used to perturb the DW images**.

Then, the pseudo ground truth diffusion datasets were perturbed by simulated EC distortions and rigid-body motion (one example of perturbations are shown in Figure 2). To simulate slow movement, rigid-body transformation parameters that were smooth in time were randomly generated using a 4th order polynomial model in time. EC distortions were simulated by 4 affine transformations, assuming a linear relation between applied diffusion gradient and EC distortions (for derivation of the 4 affine EC distortions and discussion of the linear relationship, see Mohammadi et al., 2010).

The perturbed data were corrected using three EC and motion correction post-processing methods: (a) none, (b) ECMOCO, and (c) REMATCH. The performance of each post-processing step was assessed by calculating the mean and standard deviation for the normalized rms difference between original and post-processed data, as well as the relative improvement with respect to the perturbed data (i.e., to the data with the post-processing step “none”). Furthermore, the FA map for the original “pseudo ground-truth” and the post-processed data were calculated. To calculate the diffusion tensor indices (e.g., the FA map), two fitting methods were used here: ordinary least squares (OLS) and robust-fitting (details see Mohammadi et al., 2013a,b). The robust fitting method was used to test whether the variation in the performance of the different post-processing methods was driven by outliers (i.e., DW maps that were incorrectly registered). To quantify the difference in FA obtained from different post-processing methods, the rms difference between pseudo ground-truth FA map and post-processed FA maps were calculated, as well as the relative improvement with respect to the FA from the perturbed data.

### Analysis II: the effect of post-processing on high-resolution mean kurtosis indices

MK maps were calculated from the data acquired with protocol 1 following these different post-processing methods: (a) none, (b) Gaussian smoothing (GS), (c) msPOAS, (d) REMATCH, (e) REMATCH + GS, (f) REMATCH + msPOAS.

To qualitatively demonstrate the effect of the post-processing steps on the kurtosis tensor, the MK maps were visualized for a representative subject (subject 5) and a subject that was scanned at two sessions (two-session subject 2).

To investigate the potential introduction of bias in the kurtosis tensor indices due to post-processing, the histogram of the MK maps were calculated for each subject and post-processing method.

### Analysis III: the effect of post-processing and spatial resolution on the identification of individual neuroanatomy

Here we assessed the effect of post-processing on delineating GM from WM in MK maps. To this end, GM and WM masks were generated from the GM and WM segments based on the subject's *b* = 0 image of the original dataset and R1 maps from the MPM protocol using the new multi-channel segmentation in SPM12 (Ashburner and Friston, 2005). As a measure of image contrast, the difference between the average MK in GM and WM was calculated. Furthermore, its difference with respect to the original MK-GM-WM contrast was calculated.

The effect of SNR on the MK GM-WM contrast was investigated in more detail in the high-SNR dataset acquired with protocol 2. The data were processed with the REMATCH method. MK maps with different SNR levels were generated by using differently sized subsets of the full dataset. To generate a high-SNR MK map the full DKI data was used (denoted SNR_100_ and used as pseudo ground truth). This data had an averaged SNR of about 31 in the *b* = 0 image. Then, two MK maps with lower SNR were generated using only 66% [SNR_44_ = (2/3)^2^ × SNR] and 50% [SNR_25_ = (1/2)^2^ × SNR] of the data. Finally, the SNR_25_ data was additionally processed with msPOAS and the MK map calculated. To quantify the effect of SNR on the MK GM-WM contrast, the MK difference within the WM and GM masks was calculated.

The effect of resolution on delineating neuroanatomy was demonstrated by comparing low- and high-resolution MK maps to high-resolution MT maps. To this end, the post-processed high-resolution DKI data of subject 5 (after REMATCH + msPOAS) were down-sampled to 3 mm isotropic resolution and the MK map was estimated. This low-resolution MK map was compared to the corresponding high-resolution MK map, as well as to a high-resolution MT reference image of the same subject. Since the MT map is highly sensitive to the myelin concentration (e.g., Helms et al., 2010; Freund et al., 2013b; Callaghan et al., 2014), it served not only as a macroscopic anatomical reference but also as a microstructural reference map.

## Results

### The performance of REMATCH and comparison with ECMOCO

**Figure 3:**
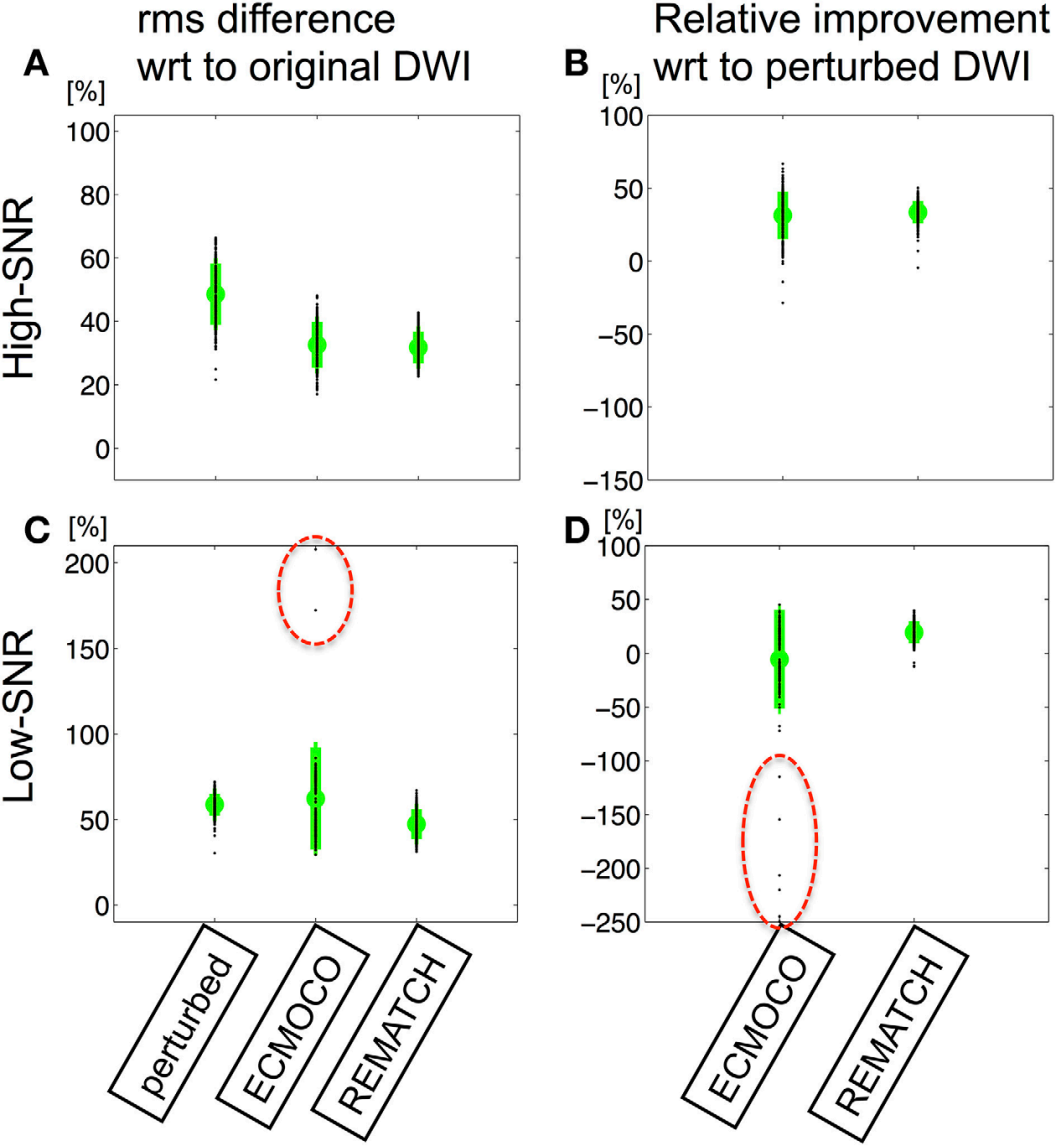
**The amount of residual misregistration is assessed by the root-mean-square (rms) difference between pseudo ground truth and perturbed DW images after post-processing: (i) no correction (perturbed), (ii) correction using ECMOCO, (iii) correction using REMATCH. (A,B)** The rms difference **(A)** and the relative **(B)** rms difference with respect to the perturbed DW maps for the high-SNR data. **(C,D)** The same as in **(A,B)** for low-SNR data. The mean and standard deviation of rms difference over DW images is depicted in green and individual rms differences are depicted as black dots. For the high-SNR data ECMOCO and REMATCH performed similarly, whereas for the low-SNR data REMATCH outperformed ECMOCO. At low SNR ECMOCO produced outliers, i.e., incorrectly registered images (black dots highlighted in **C,D**).

**Figure 4:**
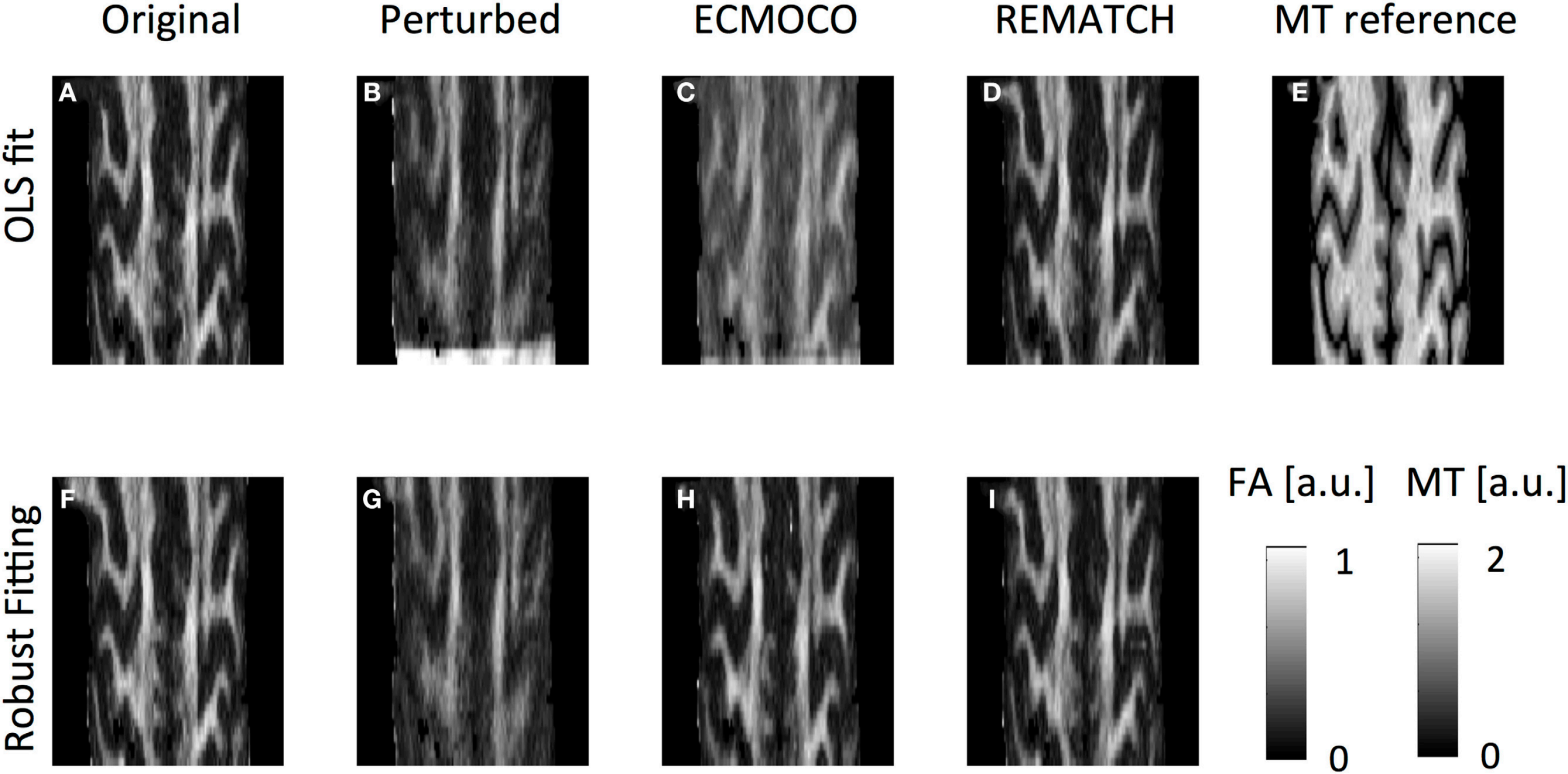
**Visual inspection of effect of misregistration on FA maps**. To calculate the FA, different datasets were used: **(A,F)** pseudo ground truth, **(B,G)** perturbed, **(C,H)** perturbed ECMOCO-processed, and **(D,I)** perturbed REMATCH-processed data. Furthermore, the diffusion tensor was fitted with different methods: **(A–D)** ordinary least squares (OLS), **(F-H)** robust fitting. **(E)** FA maps were compared with structural reference map (here: Magnetization Transfer imaging). FA map of perturbed data appeared blurrier than before and less structure was visible. After ECMOCO the FA map was biased using OLS, but the bias was removed when using robust fitting.

**Figure 5:**
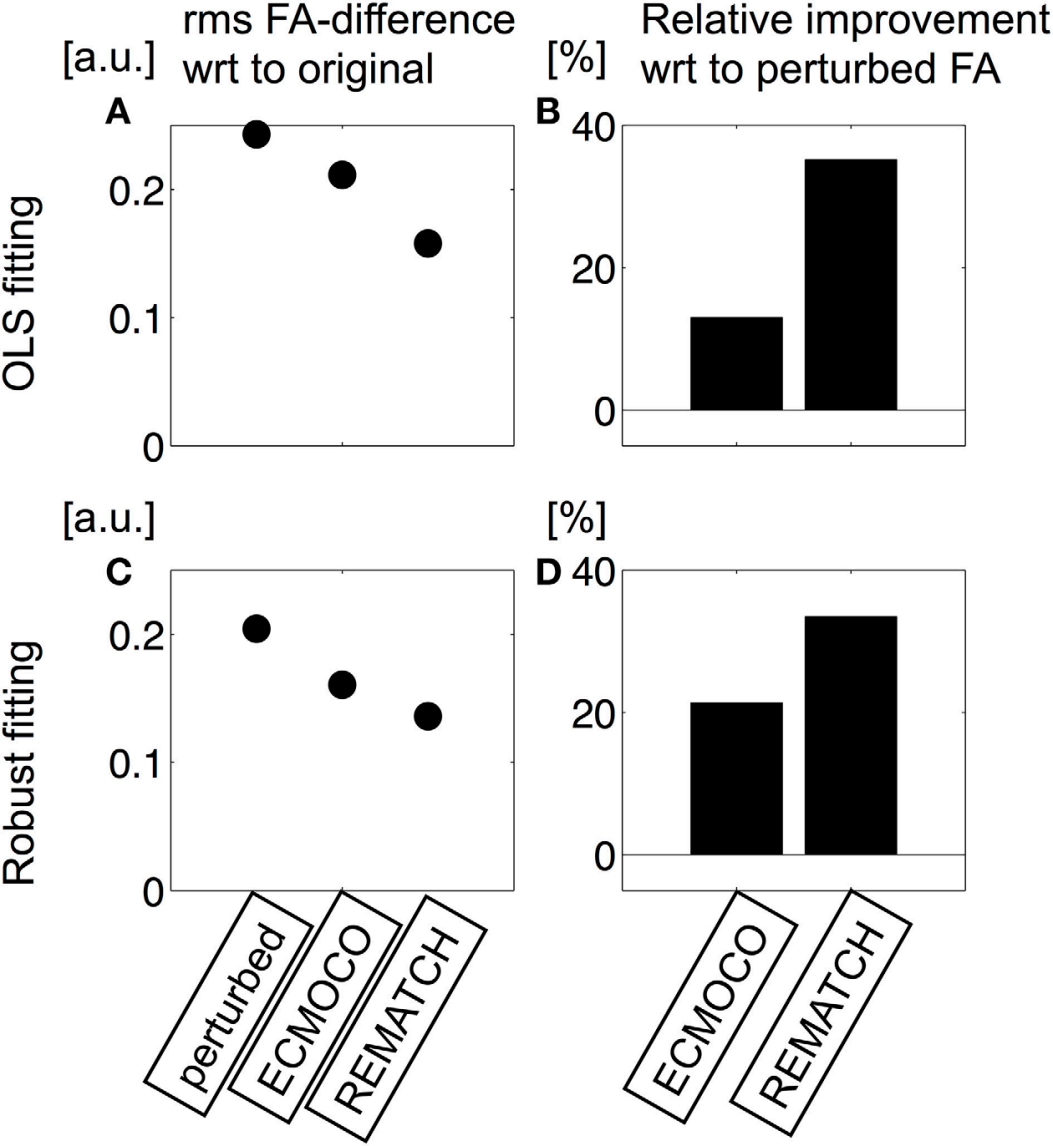
**Quantitative assessment of the effect of misregistration on FA maps (based on the same FA maps as in Figure 4)**. To this end, the rms difference in FA **(A,C)** and the relative difference **(B,D)** with respect to the perturbed FA map was calculated. If OLS fitting was used **(A,B)**, ECMOCO decreased the rms difference in FA by only 13%, whereas REMATCH decreased the rms difference by about 35%. If robust fitting was used **(C,D)**, the rms difference in FA was more similar between both methods, ECMOCO (21%) and REMATCH (33%).

To assess the performance of REMATCH, the normalized rms difference between the original and perturbed pseudo ground-truth data was calculated for each DW image and each post-processing method (high-SNR: Figures 3A,B, low-SNR: Figures 3C,D). To further investigate the effect of eddy current and motion as well as their correction on the model fit, the FA maps from the original and the perturbed low-SNR data after post-processing were visualized (Figure 4) and their difference within the brain was quantified (Figure 5). To estimate the FA maps, two different methods were used (OLS and robust fitting).

If the SNR was high, both, REMATCH and ECMOCO, improved the rms difference by more than 30% (Figure 3B). At low SNR, REMATCH still corrected about 20%, whereas ECMOCO even reduced the rms difference (about −5%, Figure 3D). More importantly, ECMOCO produced outliers at low SNR levels, i.e., incorrectly registered image (see highlighted black dots in Figures 3C,D). Note that the measure of rms difference increased with noise level and thus was not an absolute but relative measure of registration accuracy (i.e., it tells that REMATCH was relatively better than ECMOCO but not how much of the simulated image distortions were corrected).

The perturbed FA map (Figure 4B) showed less structure and appeared more blurred than the original map, especially towards the cortex (Figure 4A). The blurring was reduced by REMATCH (Figure 4D) and ECMOCO (Figure 4C). However, the FA map calculated from the ECMOCO-processed data appeared biased. As a result, the rms difference between post-processed FA and ground truth decreased substantially when REMATCH was applied as compared to ECMOCO (Figure 5A), i.e.: the FA difference was reduced by about 35% for REMATCH and 13% for ECMOCO (Figure 5B). The bias in the ECMOCO-processed FA map could be largely removed, if the tensor was fitted with robust fitting (Figure 4H), i.e., if outliers were removed. For the robust-fitted FA maps, both ECMOCO and REMATCH reduced the rms-difference in FA substantially (Figure 5C), leading to an improvement of 21% for ECMOCO and 33% for REMATCH (Figure 5D).

### The effect of post-processing on high-resolution mean kurtosis maps

**Figure 6:**
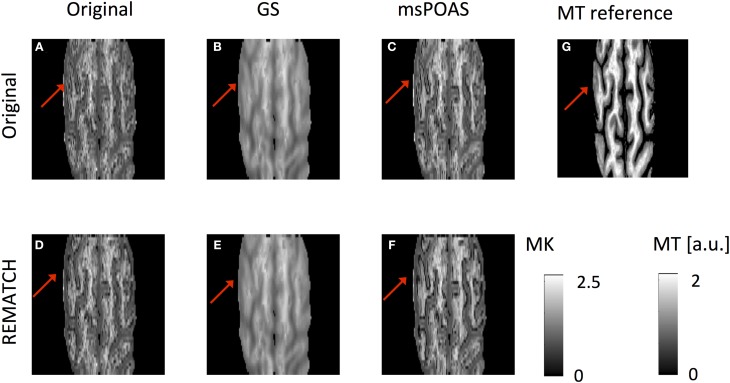
**The MK map for one representative subject after six post-processing methods: (A) none, **(B)** GS, **(C)** msPOAS, **(D)** REMATCH, **(E)** REMATCH and GS, **(F)** REMATCH, and msPOAS, as well as a high-resolution magnetization transfer (MT) map as a reference map for neuroanatomy (g)**. While GS not only denoised the data but also biased the contrast, msPAOS only denoised the MK maps. Neuroanatomical differences with respect to the MT reference image were highlighted.

**Figure 7:**
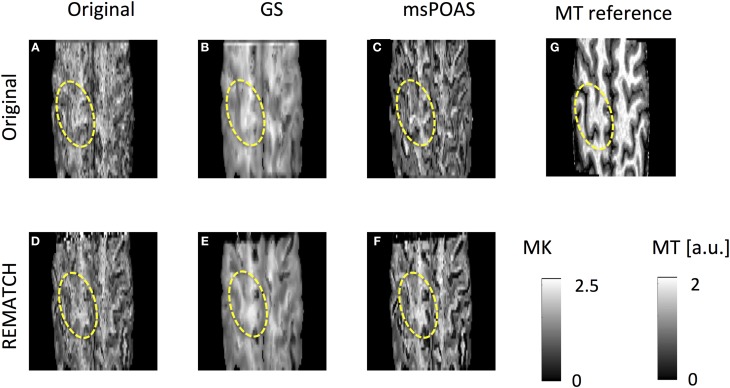
**Same as in Figure 4 for one subject, who was scanned over two sessions: (A) none, (B) GS, (C) msPOAS, (D) REMATCH, (E) REMATCH and GS, (F) REMATCH and msPOAS, as well as a high-resolution magnetization transfer (MT) map as a reference map for neuroanatomy (G)**. The resulting MK maps were altered by artifacts associated with large-scale motion, leading to neuroanatomical shape differences as compared to the MT reference map (highlighed). This bias in the MK maps could be reduced when using REMATCH.

**Figure 8:**
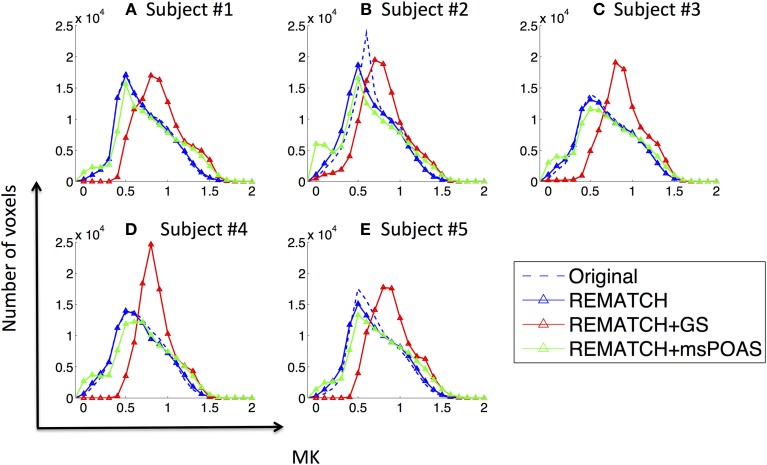
**The effect of post-processing on the distribution of mean kurtosis (MK) values within the brain for each subject (A–E)**. The original distribution had two maxima (around *MK* = 0.5 and *MK* = 1) for all subjects. Gaussian smoothing strongly changed the MK distribution, e.g., the maximum around *MK* = 0.5 was shifted to higher *MK* values. msPOAS and REMATCH changed the location of the maxima less prominently.

The effect of post-processing on the distribution of MK values within the brain was visually exemplified for two subjects [one representative subject (Figure 6) and one subject that was scanned over two sessions (Figure 7)] and summarized for each subject by its histogram (Figure 8). Visualizing the effect of Gaussian smoothing on the MK maps (Figures 6, 7) revealed that it not only denoised the data but also spuriously changed the MK values in WM and GM. The neuroanatomical shape in MK maps was altered by EC and motion related artifacts (slightly for the representative subject in Figures 6A–C and more extensively for the two-session subject in Figures 7A–C). REMATCH reduced this bias in MK maps (e.g., in Figures 6D–F and Figures 7D–F, highlighted) and thus increased the similarities with the high-resolution MT map (Figures 6G, 7G), which was used as a neuroanatomical reference map. The original MK distribution showed two maxima (Figure 8): the first one around *MK* = 0.5 and the second one around *MK* = 1. msPOAS and REMATCH left the original MK distribution unaffected and retained the two maxima. The Gaussian smoothing, however, strongly biased the MK distribution (e.g., the maximum corresponding to the *MK* = 0.5 was shifted toward *MK* = 0.8, see red curves in Figure 8). This is an indication for a systematic bias introduced by Gaussian smoothing.

### The effect of post-processing and spatial resolution on the identification of individual neuroanatomy

**Figure 9:**
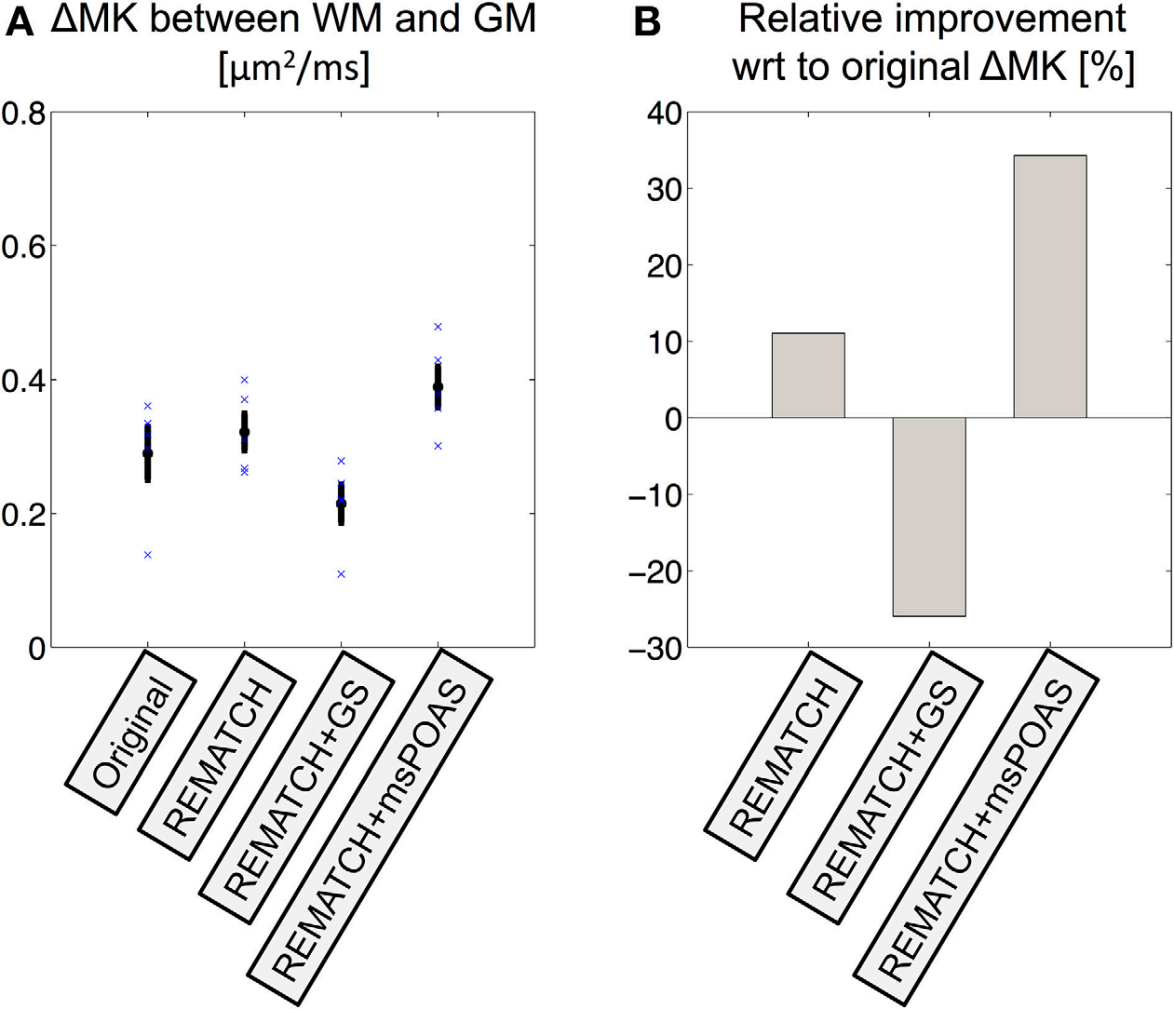
**The effect of post-processing on the MK contrast between GM and WM. (A)** The MK contrast was quantified for each subject (blue crosses) and at group level (mean: black circle, standard-error-of-the-mean: black error bars) by calculating the difference between MK in the GM and WM. **(B)** The relative improvement of MK contrast with respect to the original data. The combination of REMATCH and msPOAS increased the contrast between GM and WM by about 35%.

**Figure 10:**
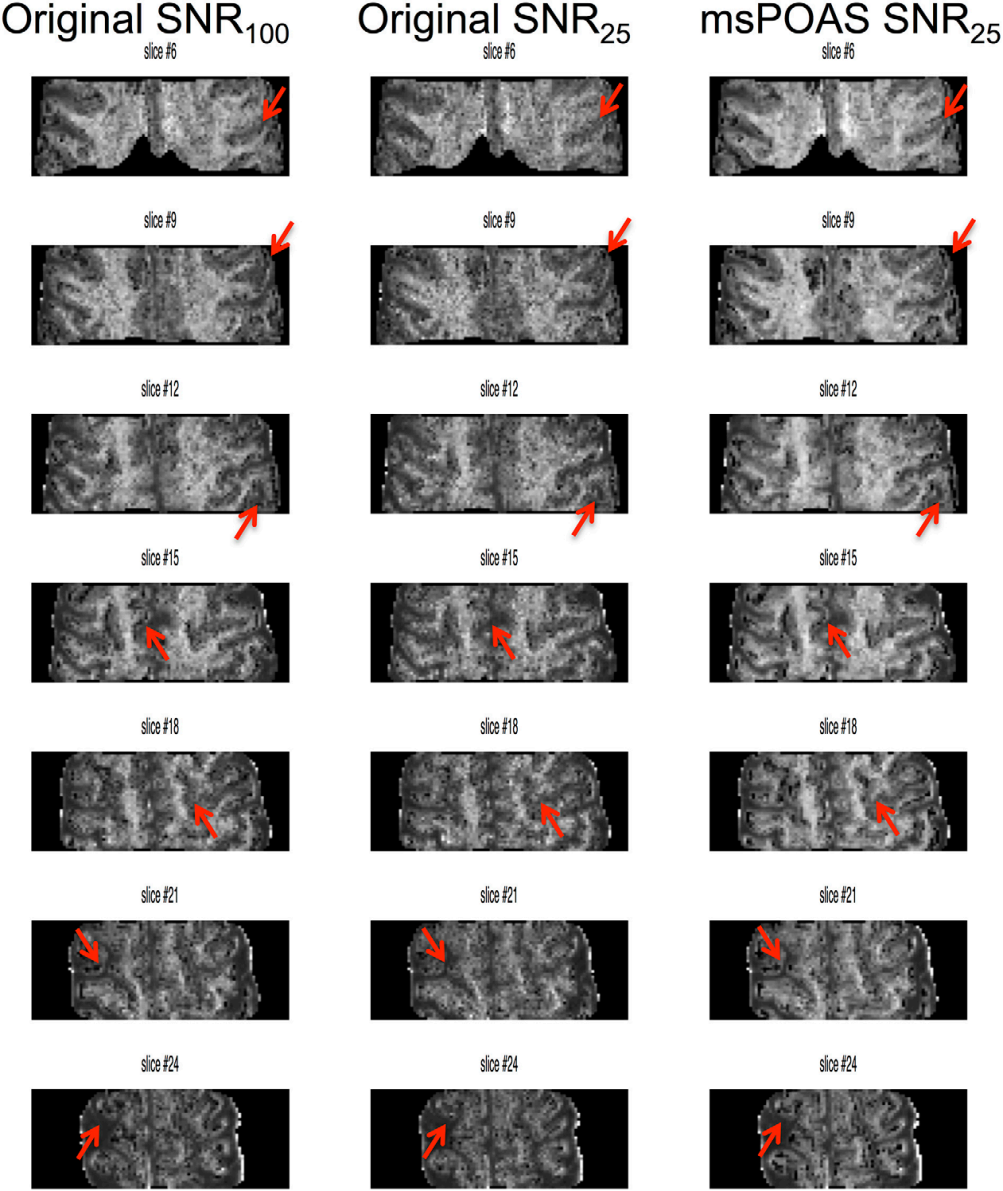
**Visual assessment of the effect of noise on MK maps**. The MK map that was calculated from the full dataset (SNR_100_) was used as pseudo ground truth and compared to MK maps, which were calculated with 50% of the original data (original SNR_25_) and 50% of the denoised data (msPOAS SNR_25_) data. Arrows highlight tissue boundaries, which were less distinctive for low-SNR data and better after processing with msPOAS.

**Figure 11:**
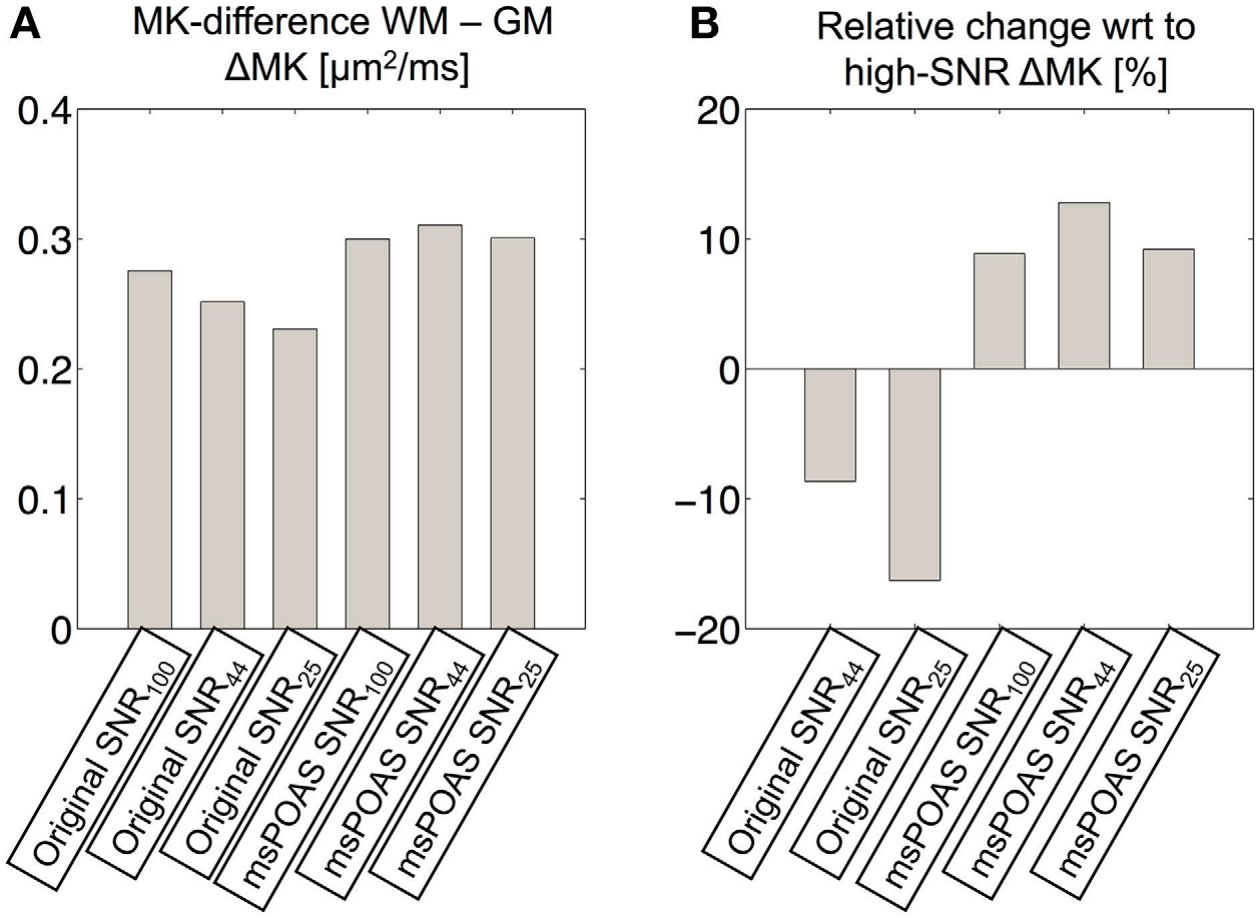
**Quantitative assessment of the effect of noise on the WM/GM MK contrast. (A)** To this end, the difference between the mean MK value within WM and GM was calculated for MK maps obtained from: all (original SNR_100_), 66% (original SNR_44_), and 50% (original SNR_25_) of the data. The same contrast was calculated after applying msPOAS on each subset: all (msPOAS SNR_100_), 66% (msPOAS SNR_44_), and 50% (msPOAS SNR_25_) of the data. **(B)** Furthermore, the relative difference with respect to ΔMK from the original SNR_100_ dataset was calculated. If less data and no denoising were employed to estimate the MK, the contrast between WM and GM was reduced. If msPOAS was used the contrast stayed approximately the same.

The effect of post-processing on the contrast between WM and GM was captured by the difference between the averaged MK in each of the two tissue segments (Figures 9–**11**). At the group level (Figure 9A), the MK-GM-WM contrast was: ΔMK = 0.29 ± 0.04 (for the original data), ΔMK = 0.32 ± 0.03 (after employing REMATCH), ΔMK = 0.22 ± 0.03 (after REMATCH and Gaussian smoothing), ΔMK = 0.39 ± 0.03 (after REMATCH and msPOAS). The improvement of the GM-WM contrast (Figure 9B) with respect to the original data was: 11% (REMATCH), −22% (REMATCH and Gaussian smoothing), and 34% (REMATCH and msPOAS).

With decreasing image SNR the MK maps became noisier and the delineation of tissue-boundaries became more challenging (arrows in Figure 10); msPOAS helped to recover some of these anatomical structures. Furthermore, with decreasing SNR the WM-GM contrast in MK maps decreased (by up to 15%), whereas if msPOAS was used the contrast increased (by about 10%, Figure 11). Furthermore, if msPOAS was used the GM-WM contrast remained almost the same independent of the decreased SNR level.

**Figure 12:**
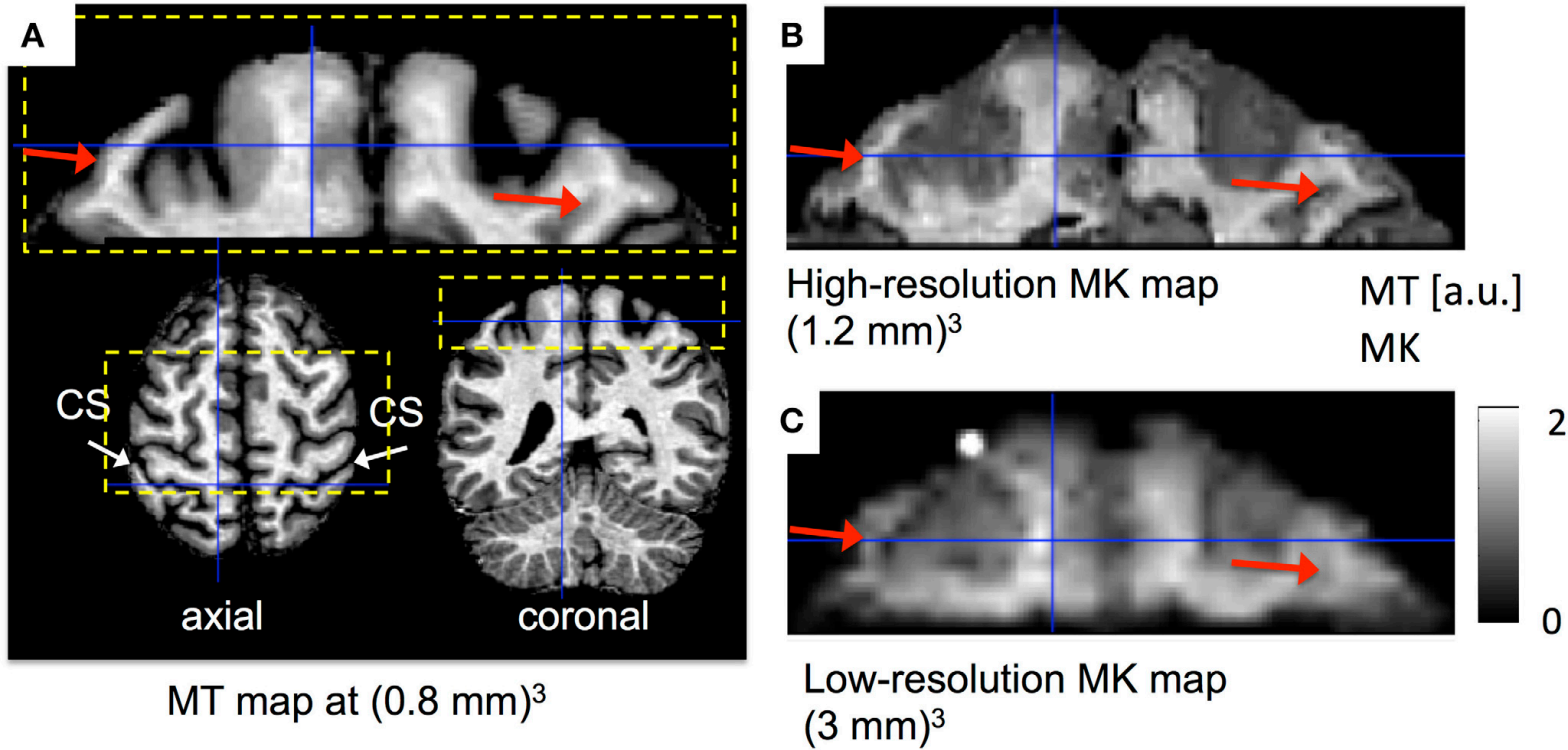
**The effect of spatial resolution on delineating neuroanatomy using MK maps. (A)** High-resolution magnetization transfer (MT) map as a reference map for neuroanatomy (the central sulcus is highlighted by the white arrows) and highly myelinated microstructure (brighter regions in the WM correspond to higher myelination). **(B,C)**: Restricted field of view (rFoV) DKI of a section of the motor cortex through the central sulcus (cs) at high **(B)** and low **(C)** spatial resolution (rFoV is highlighted in yellow, Figure 8A). The transition between highly myelinated white matter pathways and the cortical sheet (red arrows) was visible in the MK map only at high spatial resolution **(B)**.

The importance of high spatial resolution for delineating microstructural neuroanatomy is illustrated in Figure 12. Using the proposed post-processing steps (i.e., REMATCH + msPOAS), high-resolution DKI revealed highly myelinated WM fibers (red arrows) entering the motor cortex, which were not visible on the standard low-resolution DKI. To identify the degree of myelination, the corresponding MT map was used (Figure 12A).

## Discussion

We introduced three advanced post-processing methods to enable and demonstrate the advantages of high-resolution DKI. First, rFoV imaging was used to acquire two diffusion shells with 1.2 mm isotropic spatial resolution within an acceptable clinical scan time (~22 min.). Second, a novel method for Retrospective Eddy current and Motion ArtifacT Correction in High-resolution dMRI (REMATCH) was introduced. Third, Multi-Shell Position-Orientation Adaptive Smoothing (msPOAS) was employed to increase the SNR. We demonstrated that large-scale movements can bias MK maps and that this bias can be minimized using REMATCH. We found that Gaussian smoothing systematically biases the distribution of MK values within the brain, whereas msPOAS does not. We showed that the contrast between GM and WM was substantially increased when using REMATCH and msPOAS for high-resolution DKI. We illustrated that these improvements allow for high quality high-resolution DKI, which can delineate highly myelinated fibers entering the motor cortex.

DKI is related to the intra-axonal volume fraction in WM (e.g., De Santis et al., 2012) and sensitive to the neurite density in GM (e.g., Jespersen et al., 2010). Thus, MK values are expected to significantly differ between GM and WM. However, standard DKI studies have a voxel size of 2–3 mm isotropic resolution, making the delineation of GM and WM boundaries difficult. We demonstrate for the first time that DKI with 1.2 mm isotropic is possible on a standard clinical 3T scanner, although an increase in resolution from typical 2 to 1.2 mm isotropic leads to about 85% SNR reduction. Only the appropriate combination of rFOV acquisition and post-processing methods allows to compensate for this significantly reduced SNR.

High-resolution DKI can improve the delineation of GM and WM boundaries, provided the data quality is sufficiently good. We demonstrated that the contrast between GM and WM increases with increasing SNR. We introduced two post-processing methods (the combination of REMATCH and msPOAS) that increased the contrast between GM and WM by about 35%. We demonstrated the gain in microstructural information from DKI when increasing the spatial resolution. To this end, we showed that the transition between highly myelinated fibers and the cortical sheet were not visible in standard low-resolution DKI but became visible when using the proposed high-resolution DKI approach (Figure 12). This additional information will be an important step toward exploring the microstructure of the cortical sheet. This is in line with previous studies showing that the specificity of diffusion measures increases with increased spatial resolution (e.g., Roebroeck et al., 2008; Heidemann et al., 2012). For example at a voxel edge length of around one millimeter a change in the principal diffusion direction can be observed between the motor and sensorimotor cortex (e.g., see McNab et al., 2013).

The ability to generate unbiased and robust DKI measure could improve the explanatory power of clinical and neuroscience research (e.g., Hui et al., 2012; Van Cauter et al., 2012). To do this end, two approaches can be taken: (a) given the data, sophisticated fitting methods can be used to better constrain the poorly conditioned DKI model, and (b) given the fitting method, the data quality can be improved via advanced post-processing methods and imaging techniques. While a lot of studies focused on improving the model fitting (e.g., Tabesh et al., 2011; Veraart et al., 2011), currently little has been done to improve data quality. Usually single-target-based registration-approaches [e.g., eddy_correct in FSL (Jenkinson et al., 2012) or ECMOCO in ACID (Mohammadi et al., 2010)] are used for EC and motion correction, although it is known that these are less efficient at low SNR levels and high *b*-values (see e.g., Nam and Park, 2011; Ben-Amitay et al., 2012). Previous studies suggested using simulated DW images as targets, which are denoised and have a more similar contrast to the source images (e.g., Nam and Park, 2011; Ben-Amitay et al., 2012). However, these approaches only address the SNR problem for the target image but not for the source image. Furthermore, they either neglect EC distortions entirely (e.g., Ben-Amitay et al., 2012, p.) or use transformations (e.g., Nam and Park, 2011) that are independent of the expected EC distortions. As a result, the estimated distortion parameters cannot be interpreted and might even introduce additional bias (because they correct for none-existing distortions). The three main innovations in REMATCH are: (i) as opposed to previous techniques of its kind REMATCH uses a physically-informed nine-parameter-affine transformation model that specifically targets rigid-body motion and linear eddy current distortions, which can be related to the induced EC (for details see Mohammadi et al., 2010), (ii) it is the first method that combines adaptive smoothing and eddy current and motion corrections to improve alignment of low-SNR dMRI data, (iii) REMATCH provides the possibility to correct for slow and smooth subject movements directly from *b* = 0 images (option 1 in step 2). This latter option has the advantage that the rigid-body parameters estimated from *b* = 0 images correspond better to the actual subject motion and the estimated rotation parameters can be used to correct the b-matrix (Leemans and Jones, 2009) without introducing correlated noise and bias. Introducing correlated noise by applying the estimated rotation parameters to the b-matrix is a problem that can be present when standard EC and motion correction methods are used to estimate the rotation parameter (e.g., Ersoz et al., 2014). These methods estimate the rotation parameters directly from each DW image, which suffers from both, EC and motion-related image distortions. However, in the presence of EC the estimated rotation-transformation parameters can be correlated with estimated EC-distortion parameters (e.g., the shearing-type of distortions). This is because rotation and shear-type of transformations appear similar to each other from a mathematical point of view, i.e., they are not orthogonal operations with respect to each other (see, e.g., Malvern, 1977). As a result, EC-induced image shear can be falsely characterized as image rotation and thus introduce a diffusion-gradient-dependent bias into the estimated rotation parameters. This kind of correlated noise can bias the diffusion-model estimates (see e.g., discussion in Mohammadi et al., 2013b).

We showed that MK maps from subject, who were scanned over more than one session, might be biased due to artifacts associated with large-scale motion and that this bias can be reduced when using REMATCH. We showed that at lower SNR, i.e., at the SNR level of high-resolution DKI data, REMATCH improves data alignment better than standard registration methods. For low-SNR diffusion data, we recommend using REMATCH, because its additional denoising step (see step 3 in Figure 1) ensures a robust performance even at very low SNR levels as encountered in high-resolution DKI.

Furthermore, our results showed that Gaussian smoothing strongly biases the MK values, whereas adaptive smoothing (i.e., msPOAS) keeps the MK-value distribution nearly the same. Thus, instead of using Gaussian smoothing, we recommend that high-resolution DKI data should be denoised with an adaptive smoothing method such as msPOAS (Becker et al., 2014).

GM DKI is particularly affected by EC and susceptibility artifacts, because: (a) eddy currents increase towards the cortex and they scale with the gradient strength, leading to a higher artifact level in DKI, (b) susceptibility artifacts are high in sub-cortical gray matter, making whole-brain, high-resolution DKI of these structures challenging. As compared to whole-brain techniques, rFoV dMRI is less affected by EC and susceptibility distortions, because of its reduced readout time (see e.g., Wheeler-Kingshott et al., 2002; Mohammadi et al., 2013a). Thus, rFoV dMRI is particularly suited for GM DKI.

The proposed high-resolution DKI methodology using the rFoV acquisition is best suited for studies with a clear hypothesis about a defined region of interest and limited anatomical coverage (e.g., stroke and the motor cortex). New developments in simultaneously acquiring multiple imaging slices (e.g., Feinberg et al., 2010; Setsompop et al., 2012) hold the potential for accelerating multi-shell diffusion imaging and thus might allow for both high-resolution and whole-brain coverage in a reasonable acquisition time.

One limitation of the current implementation of REMATCH is that it does not correct for spatial higher-order EC distortions (e.g., see Wilm et al., 2011). For the rFOV application discussed in this paper the level of EC distortions was small and thus the higher-order effects negligible. An alternative method, which can also correct for higher-order EC distortions, is the “eddy” tool in FSL (Andersson et al., 2012). This tool, however, requires special (and usually lengthy) type of measurements, either: (a) a set of diffusion-encoding directions that span the entire sphere or (b) a blip-up-blip-down, i.e., phase encode reversed, acquisition (see http://fsl.fmrib.ox.ac.uk/fsl/fslwiki/EDDY). REMATCH does not require the special type of measurements, e.g., data that were acquired with coverage of only half of the orientation sphere and only one phase encoding blip direction will be corrected equally well. For our data the performance of the “eddy” tool was comparable to REMATCH (data not shown).

Furthermore, it should be considered that similar to other denoising methods, also msPOAS might influence the real biological variance in the data. Although msPOAS is designed to preserve the structure in the data, this requires either a sufficient contrast between intensity values or a spatial extent of homogeneous intensity structures in the space spanned by voxel locations and diffusion gradient directions. The procedure, in contrast to Gaussian smoothing, intrinsically controls a possible bias in intensity by its achieved standard deviation. In case of high SNR and small homogeneity regions msPOAS in principle leaves the original data unchanged. For details on the algorithm and further validation of msPOAS we refer to the msPOAS-methods paper (Becker et al., 2014). Despite the fact that the msPOAS intrinsically sets a limit to the probability of the occurrence of such an event, in singular voxels the deviation of the measured signal value from its expectation might be large enough to be “mistaken” as structural significant by msPOAS. Although, these “mistaken” voxels could not explain a systematic different performance of msPOAS in GM and WM, it might be argued that the strong improvement of the contrast when using msPOAS is artificially introduced. Here, we showed that the GM-WM MK contrast increased with increasing SNR even if no smoothing was applied. This is supporting evidence that the improved GM-WM contrast in MK maps after empoying msPOAS was not artificially introduced but related to successful denoising of the data.

*In-vivo* GM DKI is a relatively new field and there is currently no study published with a sufficiently high resolution that allows reporting MK values without partial volume effects in GM. We observed a previously not reported peak at very low MK values for all volunteers (Figure 9), which is mainly located at the transition between GM and CSF. We are not sure whether this peak is an artifact associated with partial volume effect at the tissue boundaries or reflects microstructure. When qualitatively comparing the MK maps at high and low SNR (Figure 10), the number of dark voxels at the edge between GM and WM, which correspond to the low MK-values in the histogram in Figure 9, increased with increasing SNR. This suggests that the low-MK values are true features of high-resolution DKI and not artifacts, which were introduced by our proposed post-processing methods.

It could also be argued that the voxel-fraction with non-Gaussian diffusion is systematically different in gray and white matter and smoothing will artificially increase this difference. However, this argument does not hold for msPOAS, because this smoothing method is not based on a specific diffusion model (e.g., DKI) but directly smoothes the DW images. We suggest that one reason for the good performance of msPOAS in increasing the GM-WM contrast is related to the spatially varying noise-profile, which is particularly pronounced in the 32-channel-head-coils data that were used in this experiment.

Finally, it should be highlighted that currently no *in-vivo* gold standard exists for dMRI measures and in particular for MK values in the brain, making an objective quality control of MK values difficult. To demonstrate the validity of our post-processing approach, we compared it to a pseudo ground truth measurement (i.e., high-resolution multi-shell dMRI with sufficient high SNR) and showed that it recovered the pseudo ground truth measurement in a recent study (see Becker et al., 2014) and in this work.

## Conclusion

We demonstrated for the first time that *in-vivo* high-quality, high-resolution DKI of the human brain is possible on a standard clinical 3T scanner if advanced acquisition and post-processing methods are used. We also showed that increasing the spatial resolution in DKI improves the delineation of the brain microstructure. We set up an imaging and post-processing pipeline and included it in an open-source software to make high-quality, high-resolution DKI more readily accessible. The proposed imaging and post-processing pipeline can also be used to generate high-quality, high-resolution diffusion maps based on other beyond-tensor models [e.g., NODDI (Zhang et al., 2012), double-wave-vector models (Lawrenz and Finsterbusch, 2013) or multi-tensor models (Behrens et al., 2003; Tabelow et al., 2012)].

### Conflict of interest statement

Siawoosh Mohammadi and Nikolaus Weiskopf have an institutional research agreement and receive support from Siemens AG. Thorsten Feiweier is an employee of Siemens AG. Thorsten Feiweier owns stocks of Siemens AG. Thorsten Feiweier holds patents filed by Siemens AG. The authors declare that the research was conducted in the absence of any commercial or financial relationships that could be construed as a potential conflict of interest.
